# Self-Rated Health Trajectories among Married Americans: Do Disparities Persist over 20 Years?

**DOI:** 10.1155/2018/1208598

**Published:** 2018-01-11

**Authors:** Terceira A. Berdahl, Julia McQuillan

**Affiliations:** ^1^Division of Research and Modeling, Center for Financing, Access and Cost Trends, Agency for Healthcare Research and Quality, 5600 Fishers Lane, Rockville, MD 20852, USA; ^2^University of Nebraska-Lincoln, 709 Oldfather Hall, Lincoln, NE 68588-0324, USA

## Abstract

The purpose of this study is to understand self-rated health (SRH) trajectories by social location (race/ethnicity by gender by social class) among married individuals in the United States. We estimate multilevel models of SRH using six observations from 1980 to 2000 from a nationally representative panel of married individuals initially aged 25–55 (Marital Instability Over the Life Course Study). Results indicate that gender, race/ethnicity, and social class are associated with initial SRH disparities. Women are less healthy than men; people of color are less healthy than whites; lower educated individuals are less healthy than higher educated individuals. Women's health declined slower than men's but did not differ by race/ethnicity or education. Results from complex intersectional models show that white men with any college had the highest initial SRH. Only women with any college had significantly slower declines in SRH compared to white men with any college. For married individuals of all ages, most initial SRH disparities persist over twenty years. Intersecting statuses show that education provides uneven health benefits across racial/ethnic and gender subgroups.

## 1. Introduction

There are well-documented disparities in health by race/ethnicity, gender, and social class in the United States. Less is known about the combined advantages and disadvantages at the intersection of race/ethnicity, gender, and social class. Questions also remain regarding whether or not initial self-rated health disparities exacerbate or diminish over time. It is important to estimate, and not assume, whether social class effects on health are similar for men and women and white and nonwhite Americans. To advance knowledge of patterns of health disparities in the United States, we estimate self-rated health (SRH) trajectories over 20 years using six waves (observations) from a panel study of married individuals who were aged 25–55 in 1980 at the time of the first interview.

## 2. Background

### 2.1. Intersectionality and Health Disparities

A growing number of researchers are calling for more nuanced analyses of health inequality to acknowledge diverse outcomes of individuals who occupy different positions in social structural hierarchies [1–3]. One useful approach is the intersectionality paradigm [4–6], which emphasizes how social structures intersect to create the axis of advantage and disadvantage in multiplicative rather than only in separate or additive ways. For example, there is evidence that the experiences of women of color cannot be adequately understood by studying race/ethnicity or gender in isolation [1, 7–9]. To assess particular policies or laws, it can be appropriate to study only one axis of inequality; but to gain a more comprehensive understanding of overall changes in health with aging, comparing subgroups created by assessing multiple axes of stratification simultaneously (e.g., social class, race/ethnicity, and gender) is fruitful [10–12].

Prior work on health disparities finds that indicators of social location (e.g., race, gender, and education) contribute to health inequality [10, 13]. Men have worse mortality than women but spend fewer years with comorbidity and disability [10, 14]. Compared to whites, racial-ethnic minorities continue to face lower life expectancy and worse health outcomes from birth until death [14]. Studies of educational disparities find that people with lower education have worse health and faster health declines with aging compared to people with higher education [15, 16]. Recent studies have examined the intersections of gender and social class or race/ethnicity and social class and find that these intersections matter for health changes associated with aging [10].

Most longitudinal studies of aging include people as young as 25 and follow people for decades in small geographic areas with little racial diversity. We know of no studies that simultaneously analyze the intersections of class, race/ethnicity, and gender for twenty years. Instead, most studies focus on older and elderly adults. The current study focuses on people who were married at the start of the study. It is possible that the health protective effects of marriage could minimize gender, education, and race/ethnicity health disparities [17, 18]. Yet, recent research highlighting the long reach of inequality from grandparents and from early life suggests that even among the married, disparities are likely to persist with aging [19–21].

### 2.2. Health Protective Effects of Marriage

Marital status is a social resource that can protect against steep health declines associated with aging. Among people who are married, the influence of depressive symptoms and chronic conditions on subsequent functional limitations is weaker compared to nonmarried [22]. The end of a marriage is also associated with higher allostatic load (an indicator of biological risk for worse health) [23]. The physiological impact of marital disruption is larger for widowhood than divorce, as indicated by comparisons of inflammatory, metabolic, and cardiovascular functioning compared to those who remain married [23].

Rates of marriage, like health disparities, vary by race and social class. Marriage rates and duration are lower among people with lower education and racial/ethnic minorities compared to people who have higher education and who are white [24]. Therefore, health differences by race/ethnicity and education could be smaller among the married than the nonmarried because of selection into a status with privileges [25]. In 1980, the difference in ever-married rates among white women (95%) and black women (87%) was smaller than that in 2012 (87% for white women and 63% for black women) [24]. Because marriage was ubiquitous in 1980, it is unclear if there will be health disparities in this more narrow group than the general population, yet we argue that it is important to find out, because marriage is sometimes promoted as a way to reduce social problems, even though there is evidence that marriage does not have the same privileges for all [26].

## 3. Current Study

In the present study, we advance understanding of health disparities by answering two important questions: In the United States, are there health disparities among people who are married that are created by disadvantaged social locations due to the intersections of gender, race, and education? If yes, do disparities persist over decades? To answer these questions, we examined self-rated health trajectories of a random sample of individuals who were married in 1980 and followed over twenty years. We restricted the sample to adults who were initially aged 25–55 years. We categorized individuals by gender (men or women), race/ethnicity (white or nonwhite), and level of education (up to less than a high school degree, a high school degree, or any level of college education), thus creating twelve subgroups with varying advantages and disadvantages.

## 4. Data and Methods

For the current study, we used data from the *Marital Instability Over the Life Course (MIOLC)* study, a six-wave panel study of a national sample of initially married persons in the United States [27]. Respondents were interviewed by telephone in 1980, 1983, 1988, 1992, 1997, and 2000 (*waves 1–6*). Only the wife or the husband was included in the study; the participant was selected by random assignment. During the first wave of the study, the survey involved random digit dialing and screening of individuals for inclusion in the study. The inclusion criteria required that individuals had to be married and between the ages of 18 and 55. Among eligible households, the rate of completed interviews for the initial wave of the study was 65%, yielding a sample size of 2,034. The response rates varied among subsequent waves of data collection (in the order: 78%, 84%, 88%, 90%, and 82%). The analytical sample for this study includes those between the ages of 25 and 55 at the initial interview in order to ensure that they were likely to have finished their education. Multilevel models incorporate data from as many waves as participants completed; as with all longitudinal studies, there was attrition over time. Comprehensive information about the data set and how well it represents the U.S. married population of 1980 and comparisons with formerly married adults in 2000 is provided in the methodology report [28].

### 4.1. Variables/Measures

#### 4.1.1. Self-Rated Health (SRH)

We measure self-rated health, the dependent variable, with the following question: “Now I have some questions about your health. In general, would you say your own health is excellent (3), good (2), fair (1), or poor (0)?” This single-item measure has a strong association with mortality, suggesting that it is a valid indicator of health [29]. Self-rated health is measured in every wave of data collection in the same way.

#### 4.1.2. Wave/Trajectory

To measure the changes in self-rated health, we include a variable that indicates the wave of observation. This variable is coded as 0 for the first interview in 1980 and 5 for the last interview in 2000, with consecutive numbers in between. There were about three years between interviews. With this variable in the model, the intercept indicates the average initial self-rated health, and the coefficient for “wave” indicates the average change in self-rated health between observations, or the trajectory of change in health. The variance component for this variable indicates the amount of spread around the average trajectory.

#### 4.1.3. Age

We included age (measured in years) as an individual level characteristic to adjust for differences in initial health status. We mean center age so that the intercept represents participants with the average age for the sample.

#### 4.1.4. Social Location

We measure social class using education, a common practice in health research [30]. We use only the initial value of education because very few people add years of education beyond age 25 in the 1980s. Education was measured in years of completed formal education. We converted years into three categories. Because a high school degree is a requirement for many jobs and a college degree is required for another set of jobs, we divided years into less than a high school degree (<12 years) (LTHS), high school (12 years) (HS), and any college (13+ years). The three-category measure of education helped to make meaningful subgroups for the intersectionality analysis by combining education with *gender* (what is your sex? male/female) and *race/ethnicity* (what race do you consider yourself to be? recoded into white and nonwhite) to create 12 total subgroups.

### 4.2. Analysis Strategy

We use multilevel models to estimate the trajectories of self-rated health. The multilevel model trajectory approach appropriately estimates the nesting of observations within individuals and attrition [31]. All models are estimated with random effects for the intercept (initial status) and slope parameters using HLM 7.03. Additionally, we control for initial age because the rate of health decline is likely to be steeper for those closer to 55 than to 25. The time variable is centered at the initial observation to facilitate meaningful interpretation of the constant (e.g., average initial self-rated health) [32].

Our analysis begins with a brief discussion of the sample in 1980 (see Tables 1 and 2). After presenting these descriptive statistics, we move to the HLM analysis. In Table 3, we present Model 1, which includes the time variable and serves as a baseline trajectory model for self-rated health. This model contains an intercept and a slope for time (year) with random effects for these parameters as well as an overall Level 1 error term. Next, we present estimates from Model 2 (Table 3) which includes a variable capturing respondent age at the first year of the survey (initial age). In Models 3–5 (Table 4), we present separate models with gender (Model 3), race (Model 4), and education (Model 5) to evaluate the effects of these characteristics on the aging trajectories without other variables in the model. These models illustrate how gender, race, and education modify self-rated health over time. Finally, in Model 4, we explore the joint effects of gender, race, and education by constructing comparative categories to capture the three-way interaction of these variables (Table 5).

A conventional interaction approach would also generate eleven coefficients with a comparison to the intercept and the need to rotate out the reference category using a “simple slope” approach to hypothesis testing to determine which groups are different from each other [33]. Yet, the conventional approach also requires solving equations to determine the total difference of each group from the others based upon the main effects, two-way interactions, and three-way interactions. We constructed indicator variables for the subgroups to provide a mathematically equivalent way to determine subgroup differences in SRH and simultaneously increase the ease of interpreting coefficients summarizing those differences [8]. We also estimated supplemental analyses by rotating the reference group to compare each subgroup (available from the author upon request). Our approach involves multiple significance tests, which in classical regression can increase the risk of Type 1 errors (finding a significant association when none exists). By using HLM with empirical Bayes estimation methods, we reduce the risk of Type 1 error [34]. There are practices for post hoc adjustments to reduce the risk of Type 1 error (e.g., Bonferroni adjustments), but there are arguments against this practice [35]. Because we also have some groups with small sample sizes (e.g., nonwhite women with less than a high school degree), we also risk making Type 2 errors (failing to find significance when there really is an association in the population). We therefore use the conventional 0.05 level of significance, focus on the size and meaning of differences, and use the significance tests as a heuristic for interpreting patterns of associations.

## 5. Results

### 5.1. Sample Descriptive Characteristics

We report sample descriptive statistics for the sample in Table 1. The mean age for the analytical sample was 37.24 (SD = 8.33) at the initial interview. Just over 10 percent of the sample is nonwhite (12%). Over half (58%) of the sample was women. Fewer participants have less than a high school degree (10%) than any college education (55%). On a scale from 0 to 3, with zero representing poor health and 3 representing excellent health, average initial self-rated health was above the midpoint (*M* = 2.26 and SD = 0.75).

In the initial survey, 1,785 participants responded to the variables in the analytical sample. Average self-rated health ranges from a low of 1.85 among nonwhite women with less than a high school degree (those with the most social disadvantages) to a high of 2.55 among white men with any college education (those with the most social advantages) (Table 2). For every race by the gender group, those with any college education are the largest group. Almost half of the sample consists of people who are white with any college education. The smallest groups are people who are nonwhite with less than a high school degree. Only nonwhite women do not have an education health gradient because those with any college education have lower self-rated health than those with a high school degree. For all other gender by race groups, higher education is associated with higher self-rated health.

### 5.2. Aging Trajectory Findings

We first describe the baseline model (no covariates; not shown in the table). The baseline model provides an estimate of the intercept and variance components. The intercept indicates that the average self-rated health across all participants and all waves is 2.252^∗∗∗^, a value that is above the midpoint of the 0 to 3 range for the variable (3 indicates excellent health). Average self-rated health varies significantly between people (the variance component for the intercept in the baseline model is 0.267^∗∗∗^, and for within people, the Level 1 error is 0.308). The variance components indicate that just under half of the variance in self-rated health is between individuals (47%, or (0.267/(0.267 + 0.307))) and just over half is within individuals over time (53%).

Model 1 in Table 3 provides an estimate of the aging trajectory in self-rated health, which is measured by the time variable (one unit is approximately three years). The trajectory measure has the value 0 for the first year, 1980, and 5 for the twentieth year, 2000. Therefore, the intercept for this model provides the average self-rated health in the first year of the study (1980). Initial self-rated health is 2.361^∗∗∗^, slightly higher (better) than the average for all years in the baseline model. The SRH trajectory coefficient indicates that, on average, self-rated health declines between interviews (*b* = −0.061^∗∗∗^). The standard deviation for self-rated health is 0.75. Therefore, over the six waves and twenty years, on average, self-rated health declines by −0.36 (−0.06 × 6), or almost half a standard deviation. The rate of decline in self-rated health varies across individuals, indicated by the significant variance component (variance components *p* value = 0.012^∗∗∗^).

To adjust for the thirty-year range of initial ages, we included an indicator of initial age in Model 2 (Table 3). Consistent with the strong association between age and health, health declines with age (*b* = −0.012^∗∗∗^), and the self-rated health aging trajectory is steeper for participants who were older at the start of the study (the coefficient is −0.001^∗^ larger for each additional year). The average age in the analytical sample is 37.24, with a standard deviation of 8.33 years. Therefore, the difference between a standard deviation below and above the mean age is 16.66 years translating into 24% of a standard deviation in initial self-rated health (−0.011 × 16.66 = −0.18/.75 = 24%). Initial self-rated health is arranged as we would expect, with those who are 25 reporting the best health and those age 55 the worst health. Twenty years later, all groups decline, but the decline is steeper for those who were initially older, resulting in larger differences between the age groups at the end of the study.

Building on the model with initial age and the self-rated health trajectory, we next separately explore if there are disparities by gender, race/ethnicity, or level of education for the initial or the aging trajectory of self-rated health (Table 4, Models 3–5). Women have lower average initial self-rated health (*b* = −0.085^∗∗^, or 12% of a standard deviation lower), yet the decline in health for women is less steep than it is for men (*b* = 0.037^∗∗∗^; therefore, the slope for women is −0.083^∗∗∗^ + 0.037^∗∗∗^ = −0.05). Women initially have worse health, but because their health declines more slowly, over time, they end up with better health than men twenty years later.

There is also an initial difference between nonwhite and white individuals (Table 4, Model 4). The race/ethnicity difference is larger than the gender effect (*b* = −0.266^∗∗∗^). The declines in self-rated health are not as steep for nonwhite compared to white individuals (white = −0.064; nonwhite = −0.064^∗∗∗^ + 0.022 = −0.040), yet this difference is not significant.

Consistent with prior research, there is a steep health gradient by education (Table 4, Model 5). Individuals with any college have the highest self-rated health (intercept = 2.462^∗∗∗^). Those with a high school level of education have about a fifth of a standard deviation lower self-rated health (*b* = −0.168^∗∗∗^), and the difference is even greater for those with less than a high school degree (*b* = −0.440^∗∗∗^). Although education has strong associations with initial SRH, level of education does not modify the rate of decline, adjusted for age. Those who start the study in their older age do have slightly but significantly steeper declines in health (age *b* = −0.001^∗^ + trajectory coefficient = −0.062^∗∗∗^; therefore, for each additional year, the rate of decline is −0.001 larger).

The separate models of gender, race/ethnicity, and education also show that none of these indicators of social status alone explain the variance in initial self-rated health or in the change in self-rated health over time, indicated by the significant variance components in all of the models.

We next examine the combined effect of all three indicators of social location simultaneously (Table 5, Model 6). Most of the subgroups differ in initial self-rated health relative to white men with any college, the reference group. All groups have negative coefficients, indicating lower initial self-rated health. Three groups do not have significantly lower self-rated health than white men with any college (nonwhite men with less than a high school level of education and nonwhite men and white women with any college). The differences in initial self-rated health for most groups persist with age because the rate of decline is not significantly different from the most privileged group (white men with any college: *b* = −0.08^∗∗∗^). Two groups, however, did have significantly slower declines (positive coefficients) in self-rated health with age: nonwhite women (*b* = 0.100^∗∗^) and white women (*b* = 0.029^∗^) with any college.

In addition to the comparisons with the most privileged group (white men who have any college education), it is useful to determine if there are significant differences among the groups with varying levels of privilege and disadvantage as indicated by gender, race/ethnicity, and education. We therefore reran the final model and rotated the comparison group until we estimated all possible comparisons (detailed results available upon request). Unlike the story from the separate gender, race/ethnicity, and education models, all women are not different from all men. White women at any level of education do not differ from less-educated nonwhite men (less than HS and HS). Education does not have the same strong association with self-rated health among nonwhite men and women; there are no education differences among those who are nonwhite, but there is an education health gradient among those who are white. White men have higher levels of self-rated health than nonwhite women but only if they have at least a high school level of education. There are no gender differences in self-rated health between white men and women who have the same level of education. Therefore, the main effects of gender, race, and education do not hold among all of the subgroups.

## 6. Discussion

Most prior research on health disparities focuses on either racial-ethnic, gender, or social class as separate forms of social stratification and focuses on older adults. Our findings provide support for the value of recognizing multiple intersecting systems of advantage and disadvantage simultaneously [1, 5, 6, 11]. For example, similar to prior studies, we find strong associations of education and health [36]. We did not observe similar effects of education, however, across self-rated health trajectories for all racial/ethnic and gender groups. For lower educated women, declines in health are similar to higher educated white men. For higher educated women, there are slower declines in self-rated health compared to higher educated white men. Thus, the rate of decline in self-rated health is conditioned by gender and education. Similar to Liang et al. we find racial differences in the intercept, or average health at the beginning of the study, with nonwhites scoring significantly worse on health measures [19]. Additionally, that study found few significant racial differences in aging trajectories over time, which is also similar to our study (although not directly comparable because they did not evaluate racial effects across gender and education) [19]. Consistent with our study findings, another study of aging among African Americans found that in older adults, self-rated health did not decline significantly over time [37].

The current study provides evidence that among married individuals, there are several initial differences in self-rated health, and for all but higher educated women, those differences persist over twenty years. Consistent with other studies, older age was associated with worse health initially and a steeper decline in health over time. Not surprisingly, for all age groups, self-rated health declined with aging. We were surprised to find that even among married individuals, there are important disparities in health and persistence in disparities with declines in aging. Therefore, neither selection into nor the protective effects of marriage eliminate the effects of structural inequality on health.

Data limitations require some caution in overgeneralizing these results. One limitation is that the study was designed to focus on married individuals. We do not have a comparison group of unmarried individuals during the same time period. We cannot generalize beyond the ever-married population in 1980. This limitation is somewhat mitigated by historical context because marriage was more common for whites and blacks in 1980 [24]. We expect that health status, however, would be worse among unmarried individuals; therefore, these results should not be generalized beyond the married [17].

The MIOLC data set has smaller sample sizes for the sample of nonwhite men and women. Because of the smaller relative sample of nonwhites, it is possible our study lacks power to find statistically significant differences for subgroups with small samples in our data (more likely to have a type 2 error). As described above, there is a risk that we will falsely claim significance when nonexists with the many comparisons involved in subgroup analysis to model intersectionality.

In a recent study, Ferarro et al. reviewed several possible mechanisms for long-standing racial and ethnic health disparities (e.g., differential exposure to environmental hazards, poverty, higher rates of smoking, more dangerous jobs, less access to health care, accumulation of disadvantages, and weathering) [3]. A central idea in health disparities research is that differences between groups constructed from different locations in social hierarchies reflect modifiable characteristics potentially amenable to interventions. Even with our limitations, the current study provides valuable evidence that for most groups, health disparities persist with age, yet for women, higher education can be protective (but not for nonwhite men). In addition, we have an unusually wide range of ages and participants are followed for two decades, providing considerable data to estimate health trajectories. Many studies of aging tend to start when people are in the 50s, because most studies rely upon data sources such as the Health and Retirement Survey, which is limited to individuals aged 50 or older [19].

## 7. Conclusion

Our findings join the growing effort to provide more context for health inequality and to address groups that have been historically marginalized (e.g., women of color). This approach also allows us to compare groups that are simultaneously advantaged and disadvantaged, such as lower educated white men and women, groups recently receiving increased research and policy attention [38, 39]. As urged by Bauer, our study illustrates how using an intersectionality framework can further disparities research, especially among married individuals [2]. Future studies should evaluate whether or not health advantages for married individuals intersect with race, gender, and education to explain the effect of marital status on health. Our findings suggest that policy efforts to promote marriage are not likely to reduce health disparities, but discovering if disparities exist even among the married will add to efforts to reduce disparities by focusing efforts on factors that matter the most [26]. In addition to healthier people “selecting into” marriage, there is also evidence that marriage has health benefits [18], particularly for men [40]. However, this benefit may not extend to nonwhite men given lower marriage and higher cohabitation rates among African Americans compared to white Americans [24].

Examining multiple subgroups with varying levels of privilege and disadvantage reveals that the most structurally advantaged group did indeed have the best initial self-rated health. Most, but not all, subgroups declined in health at similar rates. Therefore, we cannot expect that health disparities will diminish with time, as would occur if age was a leveler [15, 16]. Instead, gaps persist, and therefore efforts to improve health disparities by gender, race, and class are still needed.

## Figures and Tables

**Table 1:** Sample descriptive statistics for MIOLC wave 1, aged 25–55.

	Mean (%)	SD
Self-rated health	2.26	0.75
Age (in years)	37.24	8.33
Race		
Nonwhite	11.9	—
White	88.1	—
Gender		
Men	41.5	—
Women	58.5	—
Education		
<HS	10.4	—
High school graduate	63.1	—
Any college	26.5	—

*N* = 1,785.

**Table 2:** Self-rated health by gender, race, and education for adults aged 25–55 in 1980.

			Mean	SD	*N*
Women	White	Less than high school	2.09	0.85	88
		High school graduate	2.30	0.72	371
		Any college	2.49	0.67	455
	Nonwhite	Less than high school	1.85	0.81	20
		High school graduate	2.15	0.70	52
		Any college	2.05	0.71	58
Men	White	Less than high school	1.88	1.00	57
		High school graduate	2.36	0.77	181
		Any college	2.55	0.62	420
	Nonwhite	Less than high school	2.10	0.89	21
		High school graduate	2.22	0.67	23
		Any college	2.31	0.73	39

MIOLC data, wave 1, *N* = 1,785.

**Table 3:** Models 1-2: self-rated health by age and self-rated health aging trajectory.

	Model 1			Model 2		
	*b*	SE	*p* value	*b*	SE	*p* value
Initial SRH (intercept)	2.358	0.016	<0.001	2.358	0.016	<0.001
× initial age	—	—	—	−0.011	0.002	<0.001
SRH trajectory (time slope)	−0.061	0.005	0.005	−0.061	0.005	<0.001
× initial age	—	—	—	−0.001	0.001	0.034

Random effects	SD	VC	*p* value	SD	VC	*p* value

Initial SRH intercept, *u*_0_	0.561	0.311	<0.001	0.551	0.301	<0.001
SRH trajectory slope, *u*_1_	0.111	0.011	<0.001	0.111	0.011	<0.001
Level 1, *r*	0.511	0.261	—	0.511	0.261	—

*Note.* SD = standard deviation; VC = variance component; Level 2 MIOLC *N* = 1,785.

**Table 4:** Models 3–5: self-rated health trajectories by gender, race, and education.

	Model 3			Model 4			Model 5		
	*b*	SE	*p* value	*b*	SE	*p* value	*b*	SE	*p* value
Initial SRH (intercept)	2.410	0.025	<0.001	2.389	0.017	<0.001	2.462	0.020	<0.001
× initial age	−0.012	0.002	<0.001	−0.012	0.002	<0.001	−0.010	0.002	<0.001
Women (men = reference group)	−0.085	0.033	0.009	—	—	—	—	—	—
Nonwhite (white = reference group)	—	—	—	−0.266	0.050	<0.001	—	—	—
Less than HS (any college = reference group)	—	—	—	—	—	—	−0.440	0.065	<0.001
High school	—	—	—	—	—	—	−0.168	0.034	<0.001
SRH trajectory (time slope)	−0.083	0.008	<0.001	−0.064	0.005	<0.001	−0.062	0.006	<0.001
× initial age	−0.001	0.001	0.064	−0.001	0.001	0.041	−0.001	0.001	0.020
Women (men = reference group)	0.037	0.010	<0.001	—	—	—	—	—	—
Nonwhite (white = reference group)	—	—	—	0.022	0.020	0.289	—	—	—
Less than HS (any college = reference group)	—	—	—	—	—	—	0.021	0.024	0.392
High school	—	—	—	—	—	—	−0.007	0.010	0.496

Random effects	SD	VC	*p* value	SD	VC	*p* value	SD	VC	*p* value

Initial SRH intercept, *u*_0_	0.546	0.298	<0.001	0.541	0.293	<0.001	0.532	0.282	<0.001
SRH trajectory slope, *u*_1_	0.106	0.011	<0.001	0.108	0.012	<0.001	0.108	0.012	<0.001
Level 1, *r*	0.508	0.258	—	0.508	0.258	—	0.508	0.258	—

*Note.* SRH = self-rated health; VC = variance component; SD = standard deviation; Level 2 MIOLC *N* = 1,785.

**Table 5:** Model 6: self-rated health trajectories by gender, race, and education.

	Model 6		
	*b*	SE	*p* value
Initial SRH (intercept)	2.52	0.03	<0.001
× initial age	−0.01	0.00	<0.001
Race by gender by education			
× nonwhite women LTHS	−0.67	0.14	<0.001
× nonwhite women HS	−0.42	0.08	<0.001
× nonwhite women any college	−0.47	0.09	<0.001
× nonwhite men LTHS	−0.32	0.18	0.07
× nonwhite men HS	−0.30	0.14	0.03
× nonwhite men any college	−0.22	0.12	0.06
× white women LTHS	−0.47	0.09	<0.001
× white women HS	−0.22	0.05	<0.001
× white women any college	−0.05	0.04	0.19
× white men LTHS	−0.54	0.13	<0.001
× white men HS	−0.19	0.06	0.00
SRH trajectory (time slope)	−0.082	0.010	<0.001
× initial age	−0.001	0.001	0.047
Race by gender by education			
× nonwhite women LTHS	0.057	0.128	0.653
× nonwhite women HS	0.042	0.035	0.226
× nonwhite women any college	0.096	0.035	0.006
× nonwhite men LTHS	0.064	0.070	0.361
× nonwhite men HS	−0.041	0.043	0.345
× nonwhite men any college	−0.045	0.051	0.383
× white women LTHS	0.050	0.030	0.099
× white women HS	0.022	0.014	0.105
× white women any college	0.029	0.013	0.022
× white men LTHS	0.015	0.045	0.745
× white men HS	−0.012	0.021	0.550

Random effects	SD	VC	*p* value

Initial SRH intercept, *u*_0_	0.525	0.275	<0.001
SRH trajectory slope, *u*_1_	0.106	0.011	<0.001
Level 1, *r*	0.508	0.258	—

*Note.* The reference group is white men with any college; LTHS = less than high school; HS = high school; AC = any college; Level 2 MIOLC *N* = 1,785.
